# DEVELOPMENT AND ASSESSMENT OF THE VIRTUAL INTERGENERATIONAL COMPARATIVE GLOBAL AGING CURRICULUM

**DOI:** 10.1093/geroni/igae098.3834

**Published:** 2024-12-31

**Authors:** Grzegorz Zurek, Alina Zurek, Lyn Holley

**Affiliations:** Wroclaw University of Health and Sport Sciences, Wroclaw, Dolnoslaskie, Poland; University of Wroclaw, Wroclaw, Dolnoslaskie, Poland; University of Nebraska at Omaha, Omaha, Nebraska, United States

## Abstract

This presentation describes didactic process in the international, experimental, hybrid course, Comparative Global Aging. Created by the University of Nebraska at Omaha and two Polish universities – the University of Wroclaw and the University School of Physical Education in Wroclaw, it was structured to nurture intra- and intergenerational relationships and understanding between students and older adults on different continents. Classes are conducted in a “real time” audiovisual format designed to accommodate geographical and temporal distance and cultural diversity of professors and students. Methods to track independent learning and conscious self-improvement of students were custom-fitted to the course and to the requirements of information/communication technology. The course has been conducted each year from 2018 to 2023. Qualitative data from students’ courses in each edition of the Post-Class Reflections and Learning Journals were used to evaluate and improve a series of planned and systematic teaching methods inspired by both teachers and students. Data are from 44 UNO students, 23 students from Poland and 35 students from European universities in 9 European countries participating in the ERASMUS academic student exchange program.. Although courses were conducted in English, student teams were intentionally multinational and multicultural. Qualitative evaluation of data from students informed continuous improvement of methods and resources in subsequent editions of the course, and indicate it meets accepted standards for international, intergenerational, comparative, gerontological, service-learning university and virtually synchronous pedagogies, and could provide a template for courses that give learners the opportunity to work collaboratively international teams and communicate effectively.

